# Strengthening health systems through embedded research

**DOI:** 10.2471/BLT.16.189126

**Published:** 2017-02-01

**Authors:** Abdul Ghaffar, Etienne V Langlois, Kumanan Rasanathan, Stefan Peterson, Lola Adedokun, Nhan T Tran

**Affiliations:** a Alliance for Health Policy and Systems Research, World Health Organization, avenue Appia 20, 1211 Geneva 27, Switzerland.; b Health Section, United Nations Children’s Fund, New York, United States of America (USA).; c African Health Initiative, Doris Duke Charitable Foundation, New York, USA.

Realizing the health-related sustainable development goals (SDGs) requires integrated action on system-wide challenges. To address gaps in health service delivery, we need evidence on which government agencies, research institutions, donors and civil society can act.^1^ Unless research is relevant to specific health systems, the evidence that it generates can be dismissed by policy-makers.^2^ For example, there is plenty of evidence for the effectiveness of standard interventions to prevent maternal and child deaths, but countries vary widely in the degree to which these interventions have been implemented.^3^

We argue that embedding of research in real world policy, practice and implementation is needed to strengthen health systems worldwide. Embedded research conducted in partnership with policy-makers and implementers, integrated in different health system settings and that takes into account context-specific factors can ensure greater relevance in policy priority-setting and decision-making.^4^ Collaboration between researchers, implementers and policy-makers has been shown to improve uptake of health systems research.^5^ However, in many places, prioritization and conduct of research is often done solely by academics.^6^ Health research is also largely focused on biomedical and clinical interventions, while health systems and implementation research remains underfunded globally.^7^ Often, knowledge translation is an add-on activity after the completion of research projects.

The World Health Organization’s report, *Changing mindsets: strategy on health policy and systems research,* called for the embedding of research into health systems processes.^6^ This report explained that when embedding happens, researchers and decision-makers are linked through a system in which the need for evidence to inform policy is understood by decision-makers. The Alliance for Health Policy and Systems Research (AHPSR) and the United Nations Children’s Fund (UNICEF) developed a model for implementation research that addresses research priorities identified by decision-makers and specific challenges of local health systems.^8^ In this model, policy-makers and implementers at different levels in the health system are engaged as co-investigators and are involved in all phases of a research project. The approach is meant to enhance policy-makers’ and implementers’ ownership of health systems and policy research. The collaboration is designed to prioritize research on empirical questions of local relevance, generate feasible recommendations and integrate evidence into policy-making and health system strengthening.

Policy-makers, implementers and researchers are increasingly keen to collaborate on the design and conduct of research to ensure that it contributes to health policy-making.^9^^,^^10^Since 2013, AHPSR, UNICEF and Gavi, the Vaccine Alliance, have supported 26 embedded research projects in 15 low- and middle-income countries. These projects aim to foster a better understanding of health systems implementation issues linked to maternal, newborn and child health policies and programmes.^11^ Through its African Health Initiative, the Doris Duke Charitable Foundation is also supporting embedded research that aims to enhance the performance of health systems in Africa.^12^ In these contexts, embedding research in local health systems helped address real concerns of implementers and supports action to alleviate implementation barriers.

Our experience is consistent with evidence showing that embedded research facilitates the integration of scientific findings in policy implementation and health systems strengthening.^13^ However, few resources are available to support this approach. We advocate for the embedding of locally-relevant and demand-driven research in health systems worldwide to improve the implementation and scale-up of health policies, thus contributing to achievement of the health-related SDGs.
